# Deep learning prediction of chemo-immunotherapy response using tumor perfusion ultrasound images

**DOI:** 10.3389/frai.2026.1805416

**Published:** 2026-06-12

**Authors:** Kyprianos Dimou, Floris Alexandrou, Yiannis Roussakis, Constantinos Zamboglou, Triantafyllos Stylianopoulos, Chrysovalantis Voutouri

**Affiliations:** 1 Cancer Biophysics Laboratory, Department of Mechanical and Manufacturing Engineering, University of Cyprus, Nicosia, Cyprus; 2 Department of Research and Innovation, German Oncology Center, European University Cyprus, Limassol, Cyprus; 3 AnaBioSi-Data Ltd., Nicosia, Cyprus; 4 Department of Medical Physics, German Oncology Center, European University Cyprus, Limassol, Cyprus; 5 German Oncology Center, European University Cyprus, Limassol, Cyprus; 6 Department of Radiation Oncology, University of Freiburg - Medical Center, Freiburg, Germany

**Keywords:** artificial intelligence, chemo-immunotherapy response, contrast-enhanced ultrasound imaging, convolutional neural network, deep learning, predictive biomarker, synthetic data

## Abstract

**Introduction:**

Tumor heterogeneity poses a significant challenge for predicting responses to cancer therapy, highlighting the need for the development of biomarkers to guide personalized treatment. Contrast-enhanced ultrasound (CEUS) imaging is an established method to assess tumor perfusion, which directly affects drug delivery and therapeutic efficacy, as poorly perfused tumors often limit the penetration of chemo- and immunotherapeutics.

**Methods:**

We developed a deep learning framework using CEUS imaging to predict the response of tumors to chemo-immunotherapy in murine models of breast cancer, fibrosarcoma, and melanoma. A convolutional neural network (CEUS-CNN) was trained on a dataset of 587 pre-treatment CEUS images to classify tumors as responsive, stable, or non-responsive based on RECIST version 1.1 (Response Evaluation Criteria in Solid Tumors) criteria (175 responsive cases, 136 stable, and 276 non-responsive). Additionally, synthetic data were created for the responsive and stable classes to address class disparity.

**Results:**

Our framework attained an overall test accuracy of 0.877 (0.941 for responsive, 0.615 for stable, 0.963 for non-responsive) using only real data. The addition of synthetic data led to improved model performance, with a notable impact on the previously underperforming stable class. Our strategy enhanced the predictive capability of our model, raising the average test accuracy to 0.930 (1.000 for responsive, 0.769 for stable, 0.963 for non-responsive).

**Conclusion:**

These findings support CEUS imaging as a possible imaging biomarker of response to cancer therapy and further indicate that the incorporation of synthetic data can enhance model effectiveness, particularly for underrepresented classes. Together, they highlight the potential value of integrating AI with CEUS for personalized cancer treatment strategies.

## Introduction

In the battle against cancer, it is widely acknowledged that tumors are highly heterogeneous (Jain et al., 2014). They can vary significantly, not just between different types of tumors, but also within the same tumor category, as well as within the same tumor as it progresses. Consequently, the effectiveness of conventional cancer treatments differs, with certain patients responding to a specific therapy, while others see no benefit. Therefore, a new era focused on personalized, patient-specific therapeutic strategies has been introduced, emphasizing the importance of predicting a patient’s response to therapy (Borrebaeck, 2017). Specifically, these strategies are based on identifying one or more biomarkers that define the condition of a specific tumor. Such biomarkers primarily analyze the tumor genome, but only a limited number of these has been approved for cancer prediction (Borrebaeck, 2017).

In addition to genomic analysis, certain mechanical properties of tumors could serve as promising predictive biomarkers. It is well established that certain categories of stiff, “desmoplastic” tumors, characterized by a dense extracellular matrix, are difficult to treat. Softening these tumors through pharmacological interventions has been shown to enhance their response to therapy (Jain et al., 2014; Stylianopoulos et al., 2018; Martin et al., 2020; Voutouri et al., 2023; Kalli et al., 2023). Particularly, desmoplastic tumors, such as variants of breast and pancreatic cancers as well as sarcomas, undergo tissue stiffening as they proliferate in the surrounding healthy tissue. This process is driven by the activation of fibroblasts, which excessively produce extracellular matrix components, primarily collagen and hyaluronan (Stylianopoulos et al., 2012; Voutouri and Stylianopoulos, 2018). Tumor stiffening compresses intratumoral blood vessels, impairing their function, leading to reduced blood flow/perfusion and diminishing oxygen supply (Mpekris et al., 2024; Voutouri and Stylianopoulos, 2014). Hypo-perfusion in turn, limits drug delivery to the tumor, while hypoxia promotes immunosuppression, thereby undermining the effectiveness of cancer therapy (Jain, 2014; Mpekris et al., 2020; Kalli et al., 2024; Kalli and Stylianopoulos, 2024). To address these irregularities, a method has been tested to reduce tumor stiffness by reprogramming stimulated fibroblasts. This approach aims to restore normal levels of extracellular matrix production prior to therapy, and has been explored in preclinical studies in our lab and with collaborators (Jain et al., 2014; Stylianopoulos et al., 2018; Mpekris et al., 2020; Chauhan et al., 2013; Papageorgis et al., 2017; Polydorou et al., 2017; Panagi et al., 2020; Mpekris et al., 2021; Voutouri et al., 2021; Panagi et al., 2022). This approach has already demonstrated success in clinical trials (Murphy et al., 2019), with new trials scheduled (clinicaltrials.gov identifier NCT03563248, EudraCT Number: 2022-002311-39), leading to a new class of drugs, called mechanotherapeutics, designed to modulate tumor mechanics (Sheridan, 2019). Importantly, tumor perfusion can be monitored with contrast-enhanced ultrasound (CEUS). CEUS is a minimally invasive imaging modality used for diagnostic purposes in clinical practice, particularly in oncology, cardiology, and other medical conditions (Malone et al., 2020). Particularly, this method utilizes microbubble contrast agents and ultrasound imaging to visualize blood flow and calculate tissue perfusion (Malone et al., 2020; Wilson et al., 2009). Recently, we have shown that perfusion measurements using CEUS are correlated to the efficacy of cancer therapy in murine tumor models (Voutouri et al., 2023). However, there is a need to determine complex patterns and hidden attributes that relate accurately CEUS images with treatment response, so that a robust biomarker is derived.

Deep learning models in healthcare tackle a diverse range of challenges, from cancer screening and infection monitoring to providing personalized treatment recommendations (Suganyadevi et al., 2022). The main challenge in applying deep learning to medical imaging is dealing with limited datasets and a shortage of annotated samples (Roth et al., 2016; Litjens et al., 2017; Greenspan et al., 2016; Tajbakhsh et al., 2016; Jun et al., 2016). To tackle this, researchers have shifted focus to a sophisticated form of augmentation, synthetic data generation (Frid-Adar et al., 2018). This approach refers to artificially annotated information, produced through an AI algorithm that has been trained on a real dataset (Lucini, 2022; Wang et al., 2019). It is often required when real data are inadequately available or must remain confidential due to privacy concerns or compliance regulations (Bolón-Canedo et al., 2013; Abowd and Vilhuber, 2008; Dewi et al., 2022). Particularly, the lack of clinically labeled material in the healthcare industry presents a significant challenge for vision tasks, which rely heavily on the availability of labeled samples (Dewi et al., 2022). Therefore, synthetic content has gained widespread attention (Chen et al., 2021), with numerous laboratories and companies leveraging AI algorithms to produce in large amounts (Lu et al., 2022). Undoubtedly, synthetic data generation, based on patient information, has become crucial for understanding diseases while ensuring patient confidentiality and privacy (Dahmen and Cook, 2019). Synthetic samples can be created by obtaining the statistical properties of the real data to produce new instances with comparable properties (Pezoulas et al., 2024). Based on prior research, various strategies have been introduced for producing high-quality synthetic data. However, deep learning-based methods, such as virtual autoencoders (VAEs) and numerous variations of Generative Adversarial Networks (GANs) are predominant in the literature, especially for images (Pezoulas et al., 2024). Studies show that synthetic images can enhance workflow efficiency (Chen et al., 2021), support multi-modal medical image registration (Liu et al., 2019) and improve performance in tasks like brain segmentation (Bowles et al., 2018) and breast cancer diagnosis (Rai et al., 2024). Moreover, artificially generated data can boost the accuracy of AI models for inherited retinal diseases (Veturi et al., 2023) and liver lesion classification (Frid-Adar et al., 2018). Additionally, the quantity of synthetic samples can impact dataset quality and improve early diagnosis and personalized treatment (Mahmoud et al., 2023).

Latest research has highlighted the applicability of machine learning in the analysis of CEUS data. One approach involved the development and validation of a radiomics-based deep learning CEUS model (R-DLCEUS), designed to quantitatively analyze contrast-enhanced ultrasound cines (Liu et al., 2020). While the findings are promising, the study was conducted on a relatively small sample size and did not include patients from diverse ethnic backgrounds. Another study introduced an interpretable CEUS machine learning framework for classifying focal liver lesions as benign or malignant (Turco et al., 2022). Although the proposed framework is noteworthy, the relatively small and imbalanced dataset indicates that additional validation would be valuable to support the generalizability of the model. Additional work has presented a method to differentiate benign from malignant breast tumors. This approach leverages spatial and temporal features extracted from CEUS videos and employs a linear support vector machine (Kondo et al., 2023). While the approach is of interest, the study was conducted on a relatively small, single-center dataset, which may limit the generalizability of the model to broader populations. Both linear and nonlinear machine learning techniques have also been applied to differentiate between benign and malignant breast masses using CEUS data (Varghese et al., 2022). Although the methodology is robust, the use of a relatively small and non-diverse dataset suggests that further validation would be valuable to ensure generalizability and real-world performance. In the area of focal liver lesions classification, a meta-analysis was conducted to evaluate the diagnostic performance of machine learning algorithms, including both conventional and deep learning approaches using CEUS (Campello et al., 2023). While the analysis provides a comprehensive overview, substantial heterogeneity among the included studies may influence the reliability and comparability of the pooled results. Other research efforts combined a U-Net segmentation model with a feed-forward neural network model to create an automated method for classifying liver lesions in CEUS video investigations (Mămuleanu et al., 2023). The use of CEUS imaging has also been explored for automated diagnosis of focal liver lesions using deep neural networks (Căleanu et al., 2021). Lastly, the potential for automated diagnosis of hepatocellular carcinoma has been evaluated using B-mode and contrast-enhanced ultrasound images (Mitrea et al., 2021). These evaluations employed cutting-edge machine learning methods based on convolutional neural networks. Importantly, the majority of these studies are primarily focused on classification tasks, such as distinguishing between benign and malignant lesions, rather than on the prediction of treatment response.

To this end, we investigated the use of CEUS imaging to predict the response of tumors to chemo-immunotherapy in murine tumor models. In addition, we studied the application of synthetic CEUS images for this purpose. Tumor models include breast cancer (4 T1 and E0771), osteosarcoma (K7M2), fibrosarcoma (MCA205) and melanoma (B16F10) treated with chemo-immunotherapy and mechanotherapeutics to enhance perfusion (Voutouri et al., 2023) and implemented a deep learning approach based on a convolutional neural network. A dataset of 587 CEUS images, taken before administering chemotherapy, immunotherapy or their combination, was utilized for training and assessing our framework. Additionally, we explored the use of synthetic data to evaluate their impact on improving the model’s performance. Our results indicate that CEUS images analyzed with deep learning methods can predict tumor response to chemotherapy, immunotherapy or their combination with good accuracy, with further improvements observed following the incorporation of synthetic data.

## Materials and methods

### Tumor models and treatment protocol

The *in vivo* experiments employed to train and evaluate our convolutional neural networks, were carried out in previous research from our lab (Voutouri et al., 2023; Mpekris et al., 2021; Panagi et al., 2022; Mpekris et al., 2023; Mpekris et al., 2022). We developed syngeneic orthotopic models of murine breast tumors by injecting specific quantities of 4 T1 or E0771 cancer cells into the mammary fat pads of female mice. In a similar manner, osteosarcoma, fibrosarcoma, and melanoma models were established by transplanting K7M2, MCA205, and B16F10 cells, respectively, into the flanks of male or female mice. Animal experiments were conducted in compliance with the animal welfare regulations and guidelines of the Republic of Cyprus and the European Union.

**Figure 1:**
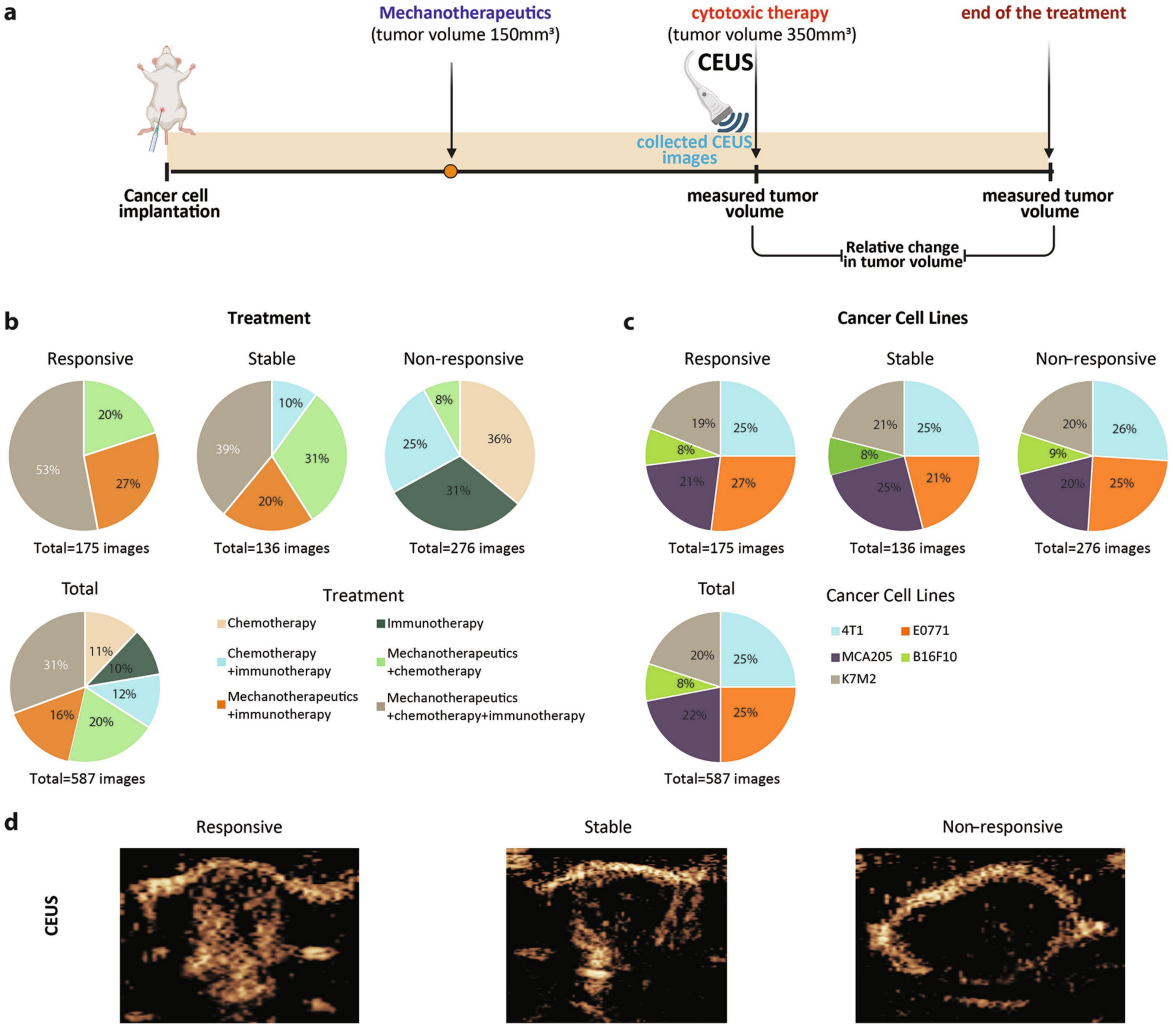
Overview of treatment protocol, classification outcomes, and imaging profiles. **(a)** Panel presents the schematic of the treatment timeline, showing administration of the mechanotherapeutic agent, chemo-immunotherapy, and tumor monitoring. **(b,c)** panels indicate the distribution of treatment responses (responsive, stable, non-responsive) by treatment type and by cancer cell line, respectively. Finally **(d)** panel shows representative contrast-enhanced ultrasound (CEUS) images for each response class.

Mice received treatment with a mechanotherapeutic agent (200 mg/kg tranilast or 500 mg/kg pirfenidone) and chemo-immunotherapy. The anti-mouse PD-L1 antibody (B7-H1, Bio X Cell, 10 mg/kg) was administered via intraperitoneal injection (i.p.), and Doxil (3 mg/kg) was delivered intravascularly (i.v.) (Voutouri et al., 2023). Treatment with the mechanotherapeutic agent was initiated once tumors attained a mean volume of approximately 150 mm^3^ (Figure 1a). When the average tumor size was measured at about 350 mm^3^, we initiated treatment with chemotherapy, immunotherapy, or their combination. Immunotherapy was administered every 3 days for three doses, while chemotherapy was given daily (Figure 1a). Tumor dimensions were frequently measured to calculate tumor volume using a digital caliper. The CEUS images were obtained when tumors in all groups reached approximately 350 mm^3^ in size, prior to the start of chemo-immunotherapy. The treatment ending was determined as the day after the administration of the third dose of immunotherapy. Tumors were categorized as responsive, stable, or non-responsive (Figures 1b,c) based on their relative volume change between the initiation of chemoimmunotherapy and the completion of treatment and in accordance with the RECIST version 1.1 (Response Evaluation Criteria in Solid Tumors) guidelines (Eisenhauer et al., 2009): responsive (≥30% tumor reduction), stable (no significant change), and non-responsive (≥20% tumor growth or new lesions). Overall, 175 cases were classified as responsive, 136 as stable, and 276 as non-responsive. Figure 1a depicts the treatment protocol followed by the experimental studies, detailing the administration timeline for the mechanotherapeutic agent, chemo-immunotherapy and tumor monitoring procedures. Additionally, Figures 1b,c present the distribution of the three classes, showing the percentages of responsive, stable, and non-responsive cases based on mode of treatment and cancer cell lines, respectively.

Ethics approval for the animal studies was granted by the Cyprus Veterinary Services committee, license number: CY/EXP/PR.L01/2024.

### Contrast—enhanced ultrasound imaging

CEUS was performed employing a Philips EPIQ Elite ultrasound system to assess tumor blood flow and perfusion parameters following an 8 μL bolus injection of the SonoVue contrast agent (Lumason in the USA) from Bracco (Milan, Italy). SonoVue is composed of sulfur hexafluoride microbubbles encapsulated in a phospholipid shell, with an average diameter of 2.5 μm. It was administered via retro-orbital injection, as according to our expertise tail vein microbubble injections are associated with high variability. Mice were anesthetized before each ultrasound session with an intraperitoneal injection of Avertin (200 mg/kg). Tumor ultrasound scanning was carried out with the L12-5 linear array transducer. Power modulation nonlinear imaging was performed at 4 MHz with a mechanical index of 0.06, using a contrast side-by-side mode (combining nonlinear bubble imaging with conventional fundamental imaging). The imaging depth was adjusted to 3 cm, with the focal point located just beneath the tumor. The gain in the bubble image was adjusted to achieve a slight level of noise throughout the image. We computed the normalized perfused area at the point when the image intensity peaks, by dividing the number of pixels with microbubble signals by the total number of pixels in the tumor region. The probe was gently positioned on the animal with excess ultrasound gel to prevent any pressure that might influence the flow. Finally, the contrast-enhanced ultrasound images were acquired when tumors in all groups attained a size of approximately 350 mm^3^, before the initiation of chemo-immunotherapy. Overall, we acquired 587 CEUS images: 175 images for the responsive class, 136 images for the stable class, and 276 images for the non-responsive class. Figure 1d illustrates representative CEUS images for each class. Importantly, each mouse contributed only a single CEUS image to the dataset.

### Pre-processing

For each image, the tumor region (Region of Interest—ROI) was manually annotated by a researcher with specialized expertise in preclinical CEUS imaging of murine tumor models. The annotator was a member of the Cancer Biophysics Laboratory at the University of Cyprus with several years of hands-on experience in tumor ultrasound acquisition and analysis. To address annotation variability, a randomly selected subset of 20% of the CEUS images (*n* ≈ 117) was independently re-annotated by a second expert. Inter-observer agreement for the delineated tumor ROIs was high, with a mean Dice similarity coefficient of 0.91 and an intraclass correlation coefficient (ICC) of 0.94 for ROI area, indicating excellent consistency between annotators. Disagreements were resolved by consensus. Regarding blinding, we emphasize an important methodological distinction specific to our preclinical setting. The response class labels (responsive, stable, non-responsive) were not assigned subjectively based on CEUS image interpretation, but were instead derived objectively from caliper-based tumor volume measurements obtained at the end of treatment, in strict accordance with RECIST version 1.1 criteria. Importantly, CEUS imaging was performed prior to the initiation of chemo-immunotherapy, whereas class labels were determined retrospectively based on post-treatment tumor volume changes. As such, the annotators delineating the tumor ROIs could not influence or bias class assignment, since labeling depended on a future, objective measurement independent of imaging-based judgment. Furthermore, to minimize any potential bias during ROI delineation, annotators were blinded to both the treatment group and the eventual response class of each animal at the time of annotation. Subsequently, the images were cropped and normalized to a fixed size to ensure compatibility with the deep learning model. This process secured that the model focused only on the relevant features while avoiding noise from the adjacent tissue.

### Deep learning models

**Figure 2:**
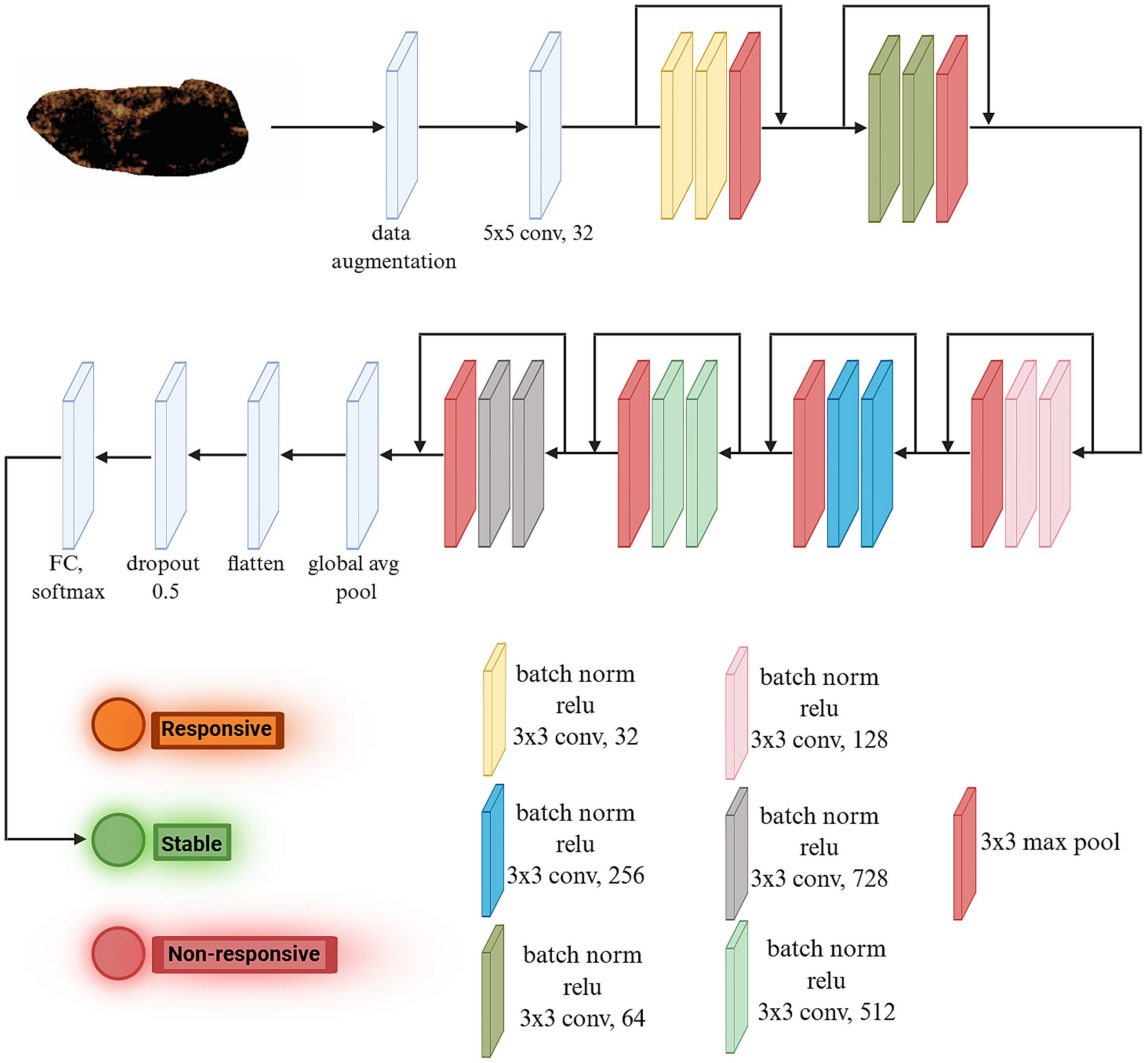
CEUS-CNN model architecture. The network processes 60 pixels (height) × 80 pixels (width) × 3 channels (RGB) CEUS images of manually outlined tumor regions. It consists of six convolutional blocks with increasing number of filters, each including batch normalization, ReLU activation, max-pooling, and residual connections. Data augmentation is applied during training. A final dropout layer and fully connected softmax layer output class probabilities.

We constructed and trained a custom convolutional neural network architecture, named CEUS-CNN (Figure 2). This architecture incorporates data augmentation techniques—including random rotation, random zoom, random brightness adjustment and horizontal flipping (Shorten and Khoshgoftaar, 2019) along with batch normalization (Ioffe and Szegedy, 2015), dropout (Srivastava et al., 2014), and residual connections (He et al., 2015). The architecture consists of six blocks. Each block is designed to progressively expand the filter count in its convolutional layers. Furthermore, each block includes batch normalization layers to enhance the model’s learning and generalization to new data, ReLU activation layers to learn complex nonlinear patterns and a max-pooling layer to downsample the feature maps and reduce the number of trainable parameters. Notably, each block concludes with a residual connection, addressing the vanishing gradient problem and facilitating the construction of deeper networks. After the six blocks, a dropout layer is added to reduce overfitting, followed by a fully connected layer with a softmax activation function to output the predicted class probabilities. The model takes as input the processed CEUS images, which display the manually outlined tumor region of interest, with dimensions of 60 pixels (height) × 80 pixels (width) × 3 channels (RGB). Prior to training, all images were normalized by dividing the pixel values by 255, effectively rescaling them to the [0,1] range and ensuring the color scales of both imaging modalities are comparable and suitable for network training. Finally, regarding the loss function we applied sparse categorical cross-entropy, while stochastic gradient descent (SGD) with a learning of 0.01 was employed as the optimizer.

Taken into account the relatively small size of the dataset, the choice of images in the training, validation and test subsets was crucial to the final performance metrics. Varying the choice of images can result in substantial fluctuations in performance metrics, with some splits producing higher scores and others considerably lower. To reduce this dependency and ensure a more reliable evaluation, we employed a 4-fold cross validation strategy in our method. Initially, 10% of the dataset was reserved exclusively as an independent test set. The remaining 90% of the data was divided into four folds and a 4-fold cross-validation scheme was applied solely for hyperparameter tuning. This approach enabled iterative training and validation across multiple fold combinations, reducing reliance on any single data split, lowering variance and enhancing the robustness of hyperparameter selection. During this stage, we implemented early stopping during training if performance on the validation dataset did not improve after 50 epochs, retrieving the best weights up to that point. This strategy ensures that the model retains its state before overfitting occurs. Subsequently, the model with the optimal hyperparameters was retained from scratch using all images included in the cross-validation procedure. The final model was then assessed on the held-out test set to evaluate its performance on entirely unseen data. Performance metrics—including sensitivity, specificity, positive predictive value (PPV), negative predictive value (NPV), F1-score, receiver operating characteristic (ROC) curve, and area under the curve (AUC)—were calculated.

### Synthetic data generation

To address the class imbalance in the dataset and examine the impact of generative images on the model’s performance, we generated synthetic data to use during training. Synthetic data were specifically generated for the responsive and stable classes, as they had fewer samples than the non-responsive class. The ultimate goal was to equalize the amount of training data across all classes.

To generate synthetic data all the images from both the responsive and stable classes, excluding those reserved for the test set, were used to fine-tune the latent diffusion model for text-to-image synthesis, SDXL (Podell et al., 2023), employing the DreamBooth technique (Ruiz et al., 2022). The process of fine-tuning SDXL along with the generation of synthetic data was carried out using Python’s library Kohya-SS. A total of 90 synthetic images were created for the responsive class and 126 synthetic images for the stable class.

Using the same hyperparameter tuned model and the same training and test datasets as before, the entire process of retraining and retesting the model from scratch was repeated. However, during this implementation, the training set was augmented with synthetic responsive and stable images to balance the amount of non-responsive images in the training data. These synthetic samples were incorporated only into the training set and were excluded from the test set, ensuring that model performance metrics remained unaffected by artificially generated data during evaluation.

All experiments were conducted using TensorFlow/Keras on a Google Colab environment equipped with a T4 GPU. Due to the relatively small input size (60 × 80 × 3) and dataset size, the proposed CNN architecture was computationally efficient. Individual training runs required approximately 3–5 min depending on convergence and early stopping criteria. The 4-fold cross-validation procedure used for hyperparameter tuning required approximately 5 h in total. Final model training on the full training set and evaluation on the independent test set were completed within a few minutes, demonstrating the computational efficiency of the proposed approach.

## Results

### Diagnostic performance of the CEUS-CNN model

**Figure 3:**
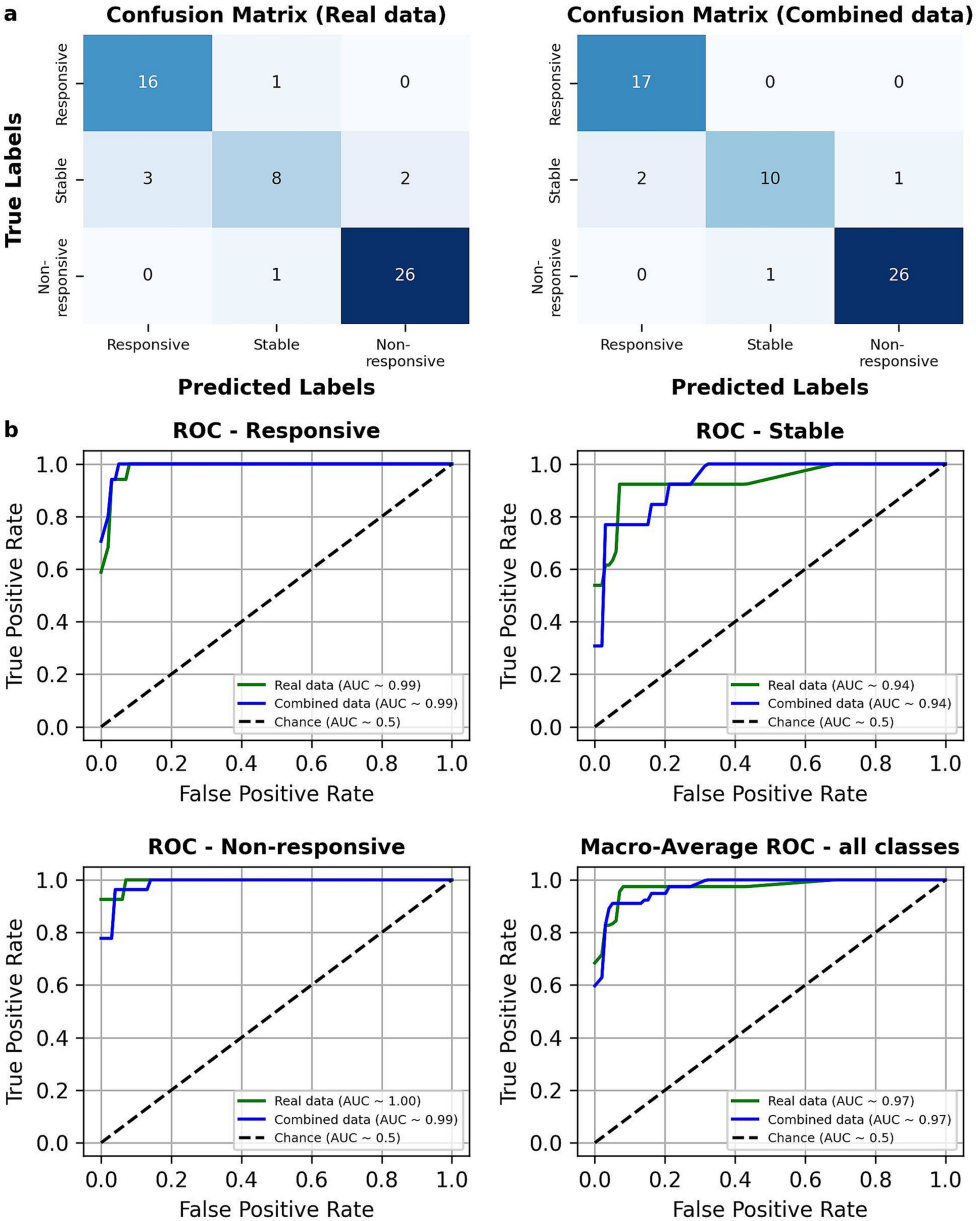
Confusion matrices, ROC curves, and AUC for the three classes. **(a)** Panel corresponds to the confusion matrices of the CEUS-CNN model, trained only with real data (left) and a combination of real and synthetic data (right). Importantly, in both cases the model did not misclassify any specimens responsive to therapy as non-responsive, and vice versa. **(b)** Panel indicates the ROC curves of the CEUS-CNN model for the responsive (top left), stable (top right) and non-responsive (bottom left) classes, trained using real data and a combination of real and synthetic data. Lastly, bottom right of panel **(b)** shows the macro-average ROC curves of the CEUS-CNN model across all classes, trained using real data and a combination of real and synthetic data. Macro-average was used instead of micro-average to ensure equal importance for all classes.

**Figure 4:**
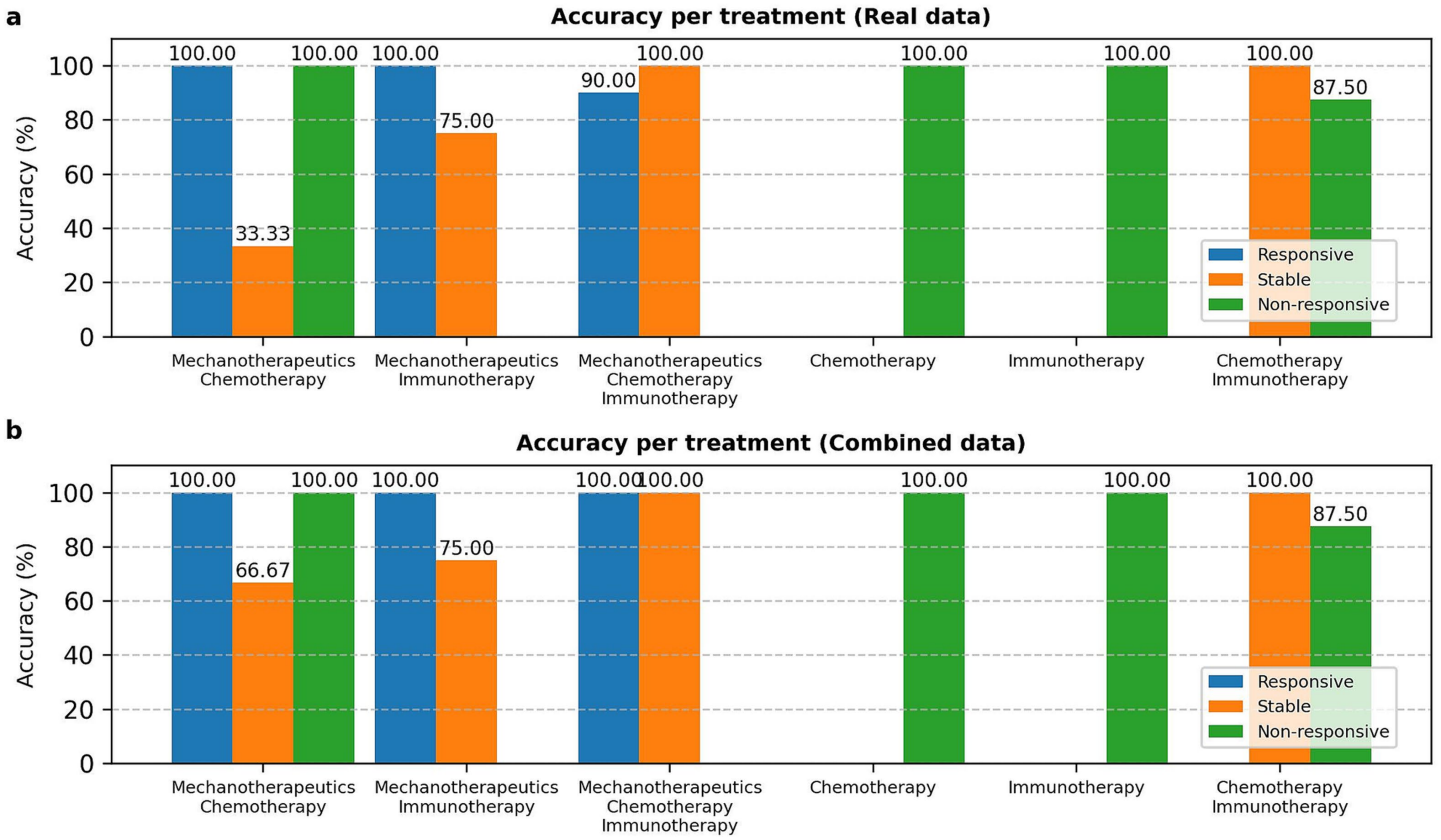
Performance of the CEUS-CNN model across treatment groups: **(a)** Panel presents the accuracy of the CEUS-CNN model across treatment groups using real data only. **(b)** Panel shows the accuracy of the CEUS-CNN model across treatment groups using a combination of real and synthetic data. For treatments involving mechanotherapeutics, model accuracy increased for responsive and stable predictions when trained with both real and synthetic data, compared to training with real data alone. Additionally, for treatments without mechanotherapeutics—comprising mostly non-responsive cases and very few stable cases—both classes were consistently predicted with high accuracy.

**Figure 5:**
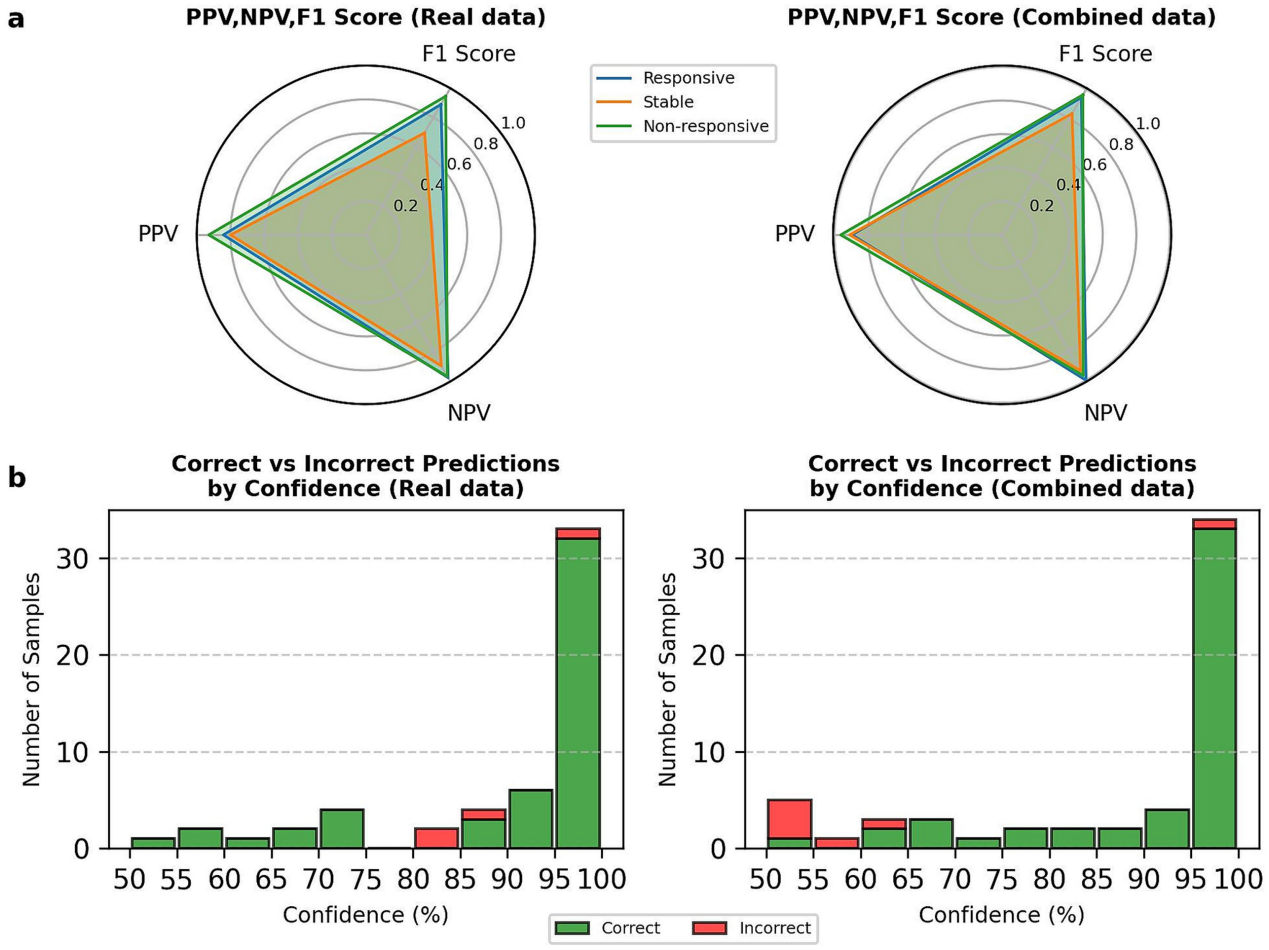
Performance metrics and prediction confidence of the CEUS-CNN model. **(a)** Panel illustrates radar plots for PPV, NPV, and F1 score for responsive, stable, and non-responsive outcomes when trained on real data (left) and on a combination of real and synthetic data (right). Training with combined data improved PPV, NPV, and F1 score for stable outcomes, while performance for responsive and non-responsive outcomes remained comparable between the two training strategies. **(b)** Panel shows the distribution of correct and incorrect predictions across confidence levels for real data (left) and combined data (right). In both cases, most predictions were made at high confidence, with the majority of high-confidence predictions being correct.

The results for the CEUS-CNN model are presented in Table 1. Overall, responsive and non-responsive classes demonstrated strong performance with respect to the evaluation metrics. On the other hand, performance for the stable class was comparatively reduced, suggesting both class imbalance and greater challenge in identifying this category. In general, the CEUS-CNN model achieved an accuracy of 0.877 on the test set. The confusion matrix of the CEUS-CNN model, shown in Figure 3a (left), indicates that the model did not misclassify any specimens responsive to therapy as non-responsive and vice versa. Additionally, Figure 3b displays the ROC curves for the three classes, assessing the same model across all thresholds. To evaluate the predictive ability of the model for every therapy, we conducted an additional analysis presented in Figure 4a. As indicated, the treatments which included mechanotherapeutics demonstrated high accuracy in predicting responsive and non-responsive outcomes. In contrast, predictions for the stable class were less accurate. Moreover, treatments without mechanotherapeutics—including mostly non-responsive cases and very few stable cases—resulted in high prediction accuracy. Finally, Figure 5a (left) illustrates a radar plot for PPV, NPV and F1 score for all classes, while Figure 5b (left) presents the distribution of correct and incorrect predictions over different confidence levels. As observed, most predictions were made with high confidence and most high-confidence predictions were correct. From a clinical perspective, these outcomes suggest that CEUS-CNN may serve as a perfusion-derived biomarker in future research, with possible relevance for supporting therapy selection and identifying patients who may benefit from mechanotherapeutic-assisted treatments.

**Table 1:** Performance metrics of the CEUS-CNN model using only real data.

Class	Sensitivity (recall)	Specificity	PPV (precision)	NPV	F1 score	Accuracy
Responsive	0.941	0.925	0.842	0.974	0.889	0.941
Stable	0.615	0.955	0.800	0.894	0.696	0.615
Non-responsive	0.963	0.933	0.929	0.966	0.945	0.963
Overall	0.840	0.938	0.857	0.944	0.843	0.877

Summary of classification performance on the test set, including sensitivity, specificity, positive predictive value (PPV), negative predictive value (NPV), F1 Score and accuracy.

### Impact of synthetic data on model performance

To address class imbalance in the dataset and assess the impact of generative images, synthetic data were created for the responsive and stable classes, which had fewer samples than the non-responsive class. The synthetic images were produced by fine-tuning the SDXL text-to-image model using DreamBooth and the Kohya-SS library, with 90 samples created for the responsive class and 126 for the stable class. The previous hyperparameter-tuned CEUS-CNN model was retrained from scratch using the same train set as in the original setup, with the addition of the responsive and stable synthetic images. The test set remained unchanged to secure an unbiased evaluation of model performance.

The results for the CEUS-CNN model using a combination of real and synthetic data are shown in Table 2. These results demonstrate that the addition of synthetic data significantly improved the model’s performance on the stable class, enhancing its ability to generalize in this previously underperforming category. Additionally, the model managed to correctly classify all responsive samples in the test set. Finally, the non-responsive class showed consistent accuracy, indicating that the inclusion of synthetic data did not adversely affect this already well-performing category. Overall, the CEUS-CNN model trained with a combination of synthetic and real data achieved an accuracy of 0.930 on the test set. The confusion matrix of the CEUS-CNN model, shown in Figure 3a (right), indicates that the model did not misclassify any specimens responsive to therapy as non-responsive, and vice versa. Importantly, using a combination of real and synthetic data improves the classification of 3 cases without introducing additional errors relative to the case using only real data. Additionally, Figure 3b presents the ROC curves for the three classes, assessing the same model across all thresholds. An additional analysis, which evaluates the performance of the model using a combination of real and synthetic data for each treatment procedure is presented in Figure 4b. Specifically, it is shown that for treatments with mechanotherapeutics, the model exhibited increased accuracy for responsive and stable predictions, compared to the case in which only real data were used during training. Regarding treatments without mechanotherapeutics, both the stable and non-responsive cases were consistently predicted with increased accuracy. Lastly, Figure 5a (right) presents a radar plot for PPV, NPV and F1 score for all classes, while Figure 5b (right) shows the distribution of correct and incorrect predictions over different confidence levels. As illustrated, most predictions were obtained with high confidence and most of high-confidence predictions were correct.

**Table 2:** Performance metrics of the CEUS-CNN model using a combination of real and synthetic data.

Class	Sensitivity (recall)	Specificity	PPV (precision)	NPV	F1 score	Accuracy
Responsive	1.000	0.950	0.895	1.000	0.944	1.000
Stable	0.769	0.977	0.909	0.935	0.833	0.769
Non-responsive	0.963	0.967	0.963	0.967	0.963	0.963
Overall	0.911	0.965	0.922	0.967	0.914	0.930

Summary of classification performance on the test set, including sensitivity, specificity, positive predictive value (PPV), negative predictive value (NPV), F1 Score and accuracy.

In summary: 1. Real-only training: high accuracy overall, but weak stable classification. 2. Real + synthetic (responsive and stable classes): improved responsive and stable accuracies without compromising the non-responsive class. This suggests that the CEUS-CNN model may benefit from synthetic augmentation and can provide useful predictive outputs that could support decision-making in future studies. Importantly, these findings reinforce the potential value of CEUS-derived perfusion features as candidate biomarkers of therapy response.

## Discussion

Undoubtedly, the application of artificial intelligence (AI) and deep learning in medical imaging and diagnostics is rapidly expanding. Our research provides strong preclinical evidence highlighting the capability of deep learning methods in leveraging CEUS data to create objective and reliable biomarkers predictive of response to cancer therapy. These biomarkers are crafted to predict tumor responses to chemo-immunotherapy, with or without the addition of mechanotherapeutics. This predictive ability is vital since it can distinguish tumors that are expected to respond to therapy versus those that are not. A key finding of our investigation is the utility of CEUS images as a mechanical/imaging biomarker. Such biomarker can offer a prediction of the tumor’s expected response prior to treatment initiation.

The CEUS-CNN model trained on real data achieved 0.877 test accuracy, without any misclassification between responsive and non-responsive cases. However, analysis across treatment groups revealed weaker performance for the stable class. To address class imbalance, synthetic data were introduced for the responsive and stable classes, increasing overall accuracy to 0.930, with improvement in the responsive class and a marked enhancement in the performance of the stable class. Notably, the performance of the non-responsive class remained consistently high.

To assess statistical significance, we applied McNemar’s test, which is appropriate for paired classification outcomes. We constructed the disagreement table at the sample level. The combined-data model correctly classified 3 samples that were misclassified by the real-data-only model, while there were no cases where the real-data-only model was correct and the combined-data model was incorrect (b = 3, c = 0). This indicates that all observed disagreements favor the CEUS-CNN model using a combination of real and synthetic data. McNemar’s test yielded a *p*-value of 0.25, which does not reach statistical significance at the conventional *α* = 0.05 level. However, we note that this result is influenced by both the very small number of discordant pairs (b + c = 3) and the limited size of the test dataset, which together reduce the statistical power of the test. In such low-disagreement and small-sample settings, McNemar’s test is inherently conservative and may fail to detect meaningful but modest performance differences, even when improvements are consistently observed. Importantly, the direction of improvement is consistent: the CEUS-CNN model using a combination of real and synthetic data improves the classification of 3 cases without introducing additional errors relative to the CEUS-CNN model using only real data. This is consistent with the observed increase in overall accuracy (0.877–0.930), suggesting a beneficial effect of synthetic data augmentation, while also highlighting the need for larger test sets to increase statistical power in future evaluations.

The underperformance of the model in classifying stable outcomes, compared to responsive and non-responsive ones, may be partly due to the smaller number of images available for this class (Ghosh et al., 2024). This data imbalance could have hindered the model’s ability to capture robust and distinctive features for the stable category. Additionally, overlapping visual characteristics between classes likely contributed to the challenge (Lango and Stefanowski, 2022). Particularly, stable samples usually display textural and color features that are partially similar to those seen in both responsive and non-responsive classes. This visual ambiguity can confuse the model, especially if the learned features are not exclusive to a single class. Moreover, the stable class may inherently represent an intermediate or transitional state, lacking distinct visual markers that strongly differentiate it from the other two categories. Such a scenario presents a challenge for the model, as it relies on clear discriminative features to accurately separate classes. Last but not least, response categories were defined strictly according to RECIST version 1.1 criteria (responsive: ≥30% reduction in tumor volume; stable: no significant change; non-responsive: ≥20% increase in tumor volume or new lesions). Within this framework, some stable cases lie near the boundaries of adjacent categories, such that small temporal variations in tumor volume may lead to reclassification. This further highlights the inherently transitional nature of the stable group. These observations suggest that the visual similarity of the stable class to both responsive and non-responsive classes may underlie its reduced classification performance. From a clinical perspective, this ambiguity underscores the need for cautious interpretation, as stable cases may represent delayed response or early resistance and could benefit from closer monitoring or the integration of additional biomarkers.

It should be noted that the primary objective of this study was not to introduce a novel deep learning architecture, but to investigate the potential of CEUS imaging as a predictive biomarker of tumor response and to assess the impact of synthetic data augmentation in the context of a limited and imbalanced dataset. For this reason, we employed a custom convolutional neural network composed of well-established components, including batch normalization, dropout, and residual connections, which was empirically optimized to provide stable and effective performance on our data.

While more advanced architectures such as ResNet or EfficientNet may offer performance benefits, their evaluation typically requires larger datasets and extensive benchmarking. To provide additional context, the proposed CEUS-CNN model was evaluated against established baseline approaches, including transfer learning with ResNet50 and EfficientNetB0 pretrained on the ImageNet dataset. When these pretrained, fine-tuned models were evaluated on the test set, they achieved an accuracy of 0.877, which is lower than the accuracy of 0.930 achieved by our proposed CEUS-CNN model. This comparison demonstrates that our custom architecture achieves improved predictive performance relative to the fine-tuned ResNet50 and EfficientNetB0 models evaluated in this study. The corresponding implementation details are available in the Supplementary material. More comparisons with established models were beyond the scope of the present study but represent an important direction for future work.

The RECIST version 1.1 criteria were originally established for clinical imaging and patient-level response evaluation. In this study, they were applied in a preclinical context as an approximate framework to ensure alignment with clinically relevant standards. Our objective is to develop a method that can ultimately assist clinical decision-making and treatment planning. Therefore, using RECIST-based categories helps maintain consistency with how treatment response is assessed in patients.

A major limitation of this study is the relatively small dataset size (587 CEUS images), derived from preclinical murine tumor models. The acquisition of such data is inherently resource-intensive, as it requires carefully controlled *in vivo* experiments and standardized imaging protocols. To mitigate this limitation, we employed a rigorous cross-validation strategy, data augmentation techniques, and synthetic data generation to improve generalization and address class imbalance. Nevertheless, larger datasets, particularly from clinical settings, will be essential to further validate the robustness and translational applicability of the proposed approach.

To assess the quality and potential bias of the synthetic data, additional evaluations were performed. A visual assessment study was conducted in which a mixed set of real and synthetic CEUS images were reviewed by two experienced clinicians. Eventually, they were unable to reliably distinguish between the two, indicating a high degree of visual realism. Furthermore, we trained the model exclusively on real data and evaluated it on a synthetic-only test set. The resulting performance was comparable to that obtained when both training and testing were performed on real data. Specifically, the model achieved an accuracy of 0.856 when trained on real data and evaluated on synthetic images, compared to 0.877 when both training and testing were conducted on real data. This suggests that the synthetic images preserve the key features required for accurate classification. Importantly, as indicated from the results, the inclusion of synthetic data improved model performance, particularly for the stable class. This improvement was achieved without evidence of overfitting, supporting their utility in enhancing generalization while not introducing observable bias.

The incorporation of AI in medical imaging is not only about improving diagnostic accuracy but also about transforming patient care. With more than 2000 clinical trials currently underway, immunotherapy is revolutionizing cancer therapy. The development of predictive biomarkers provides two key benefits: protecting patients from potentially harmful therapies and allowing for the personalization of treatment for each patient. Our research highlights the capability of ultrasound-derived perfusion images as biomarkers for predicting response to combined therapies. This creates opportunities for future clinical investigations to evaluate the potential of ultrasound or magnetic resonance imaging–based biomarkers for predicting patient responses to cancer immunotherapy.

While the present study is based on preclinical murine tumor models, we acknowledge that physiological differences between mice and humans—such as variations in hemodynamics, tumor microenvironment, and drug metabolism—may limit the direct translation of learned features to clinical populations. Therefore, the proposed framework should be interpreted as a proof-of-concept demonstrating the capability of contrast-enhanced ultrasound (CEUS)-derived perfusion as a predictive biomarker of therapy response. Importantly, while our strategy shows potential in predicting tumor responses, moving from pre-clinical models to clinical implementation presents considerable challenges. The complexity of clinical diagnosis and treatment requires a patient-specific approach that takes into account more than just tumor size. Acknowledging this, we are progressing our investigation with clinical studies at the German Oncology Center (Limassol, Cyprus) for patients with breast and prostate cancer. The goal of these studies is to verify the applicability of our model in the clinical setting. By incorporating CEUS imaging into standard diagnostic procedures, the model could potentially be adapted to account for the heterogeneous characteristics of human tumors, which may support its future applicability in guiding treatment selection. This attempt highlights our dedication to connect preclinical studies with patient care, stressing the value of multidisciplinary collaboration in introducing innovative diagnostic tools into clinical use.

To achieve an effective clinical implementation, various essential actions must be taken. These include conducting validation studies with human subjects to verify our model’s effectiveness in a clinical setting, incorporating our outcomes into current diagnostic protocols to improve tumor characterization accuracy, and establishing clear guidelines for interpreting contrast-enhanced ultrasound (CEUS) imaging results to aid in clinical decision-making. Additionally, we acknowledge the impact of tumor misclassification in clinical practice, understanding that errors in classification can significantly affect treatment planning and patient outcomes. Incorrect tumor classification could result in unsuitable treatment strategies, potentially compromising treatment effectiveness and negatively impacting the patient’s well-being. Consequently, reducing the percentage of misclassification is essential for enhancing therapeutic results and guaranteeing that patients obtain optimal care through precise tumor characterization.

We have created a deep-learning model capable of categorizing tumors into three groups according to their predicted response to treatment: responsive, stable, or non-responsive. This classification is based on the mechanical biomarker extracted from the CEUS images. This method combines the advantages of a mechanical biomarker with the predictive power of deep learning models, thus simplifying decision-making for personalized therapeutic plans. It is important to emphasize that although the mechanical properties of the tumor microenvironment, such as tumor perfusion and stiffness, are essential, biological factors are also crucial in cancer proliferation, progression, and therapy resistance. As a result, approaches like the one presented here could work in conjunction with biological markers and be taken into account by oncologists when selecting the most appropriate treatment plan.

The use of CEUS imaging for predicting tumor response, combined with the CEUS-CNN model, represents a promising complement to conventional imaging techniques. In contrast to CT or MRI, which measure tumor response according to size modifications, CEUS assesses the mechanical properties of tissues, providing a unique biomarker for treatment outcomes. This distinction highlights the potential value of CEUS in oncology, implying that it could offer more refined and possibly earlier markers of therapy effectiveness. The combination of CEUS images with cutting-edge AI models has the potential to optimize patient-specific cancer therapy, emphasizing the vital usage of novel imaging methods. In this regard, CEUS is an accessible imaging technique that might, following further validation, be considered for integration into standard diagnostic imaging practices. As a result, from the moment a tumor is first detected using ultrasound imaging, CEUS images could potentially be captured and analyzed to predict the tumor’s response to treatment. This prediction could possibly be combined with additional pertinent data that healthcare professionals use, to contribute to clinical decisions and assist in the development of optimal treatment protocols. Although our method shows promise as a tool for predicting tumor response, extensive clinical validation and further optimization will be required before potential translation into clinical use.

## Data Availability

The raw data supporting the conclusions of this article will be made available by the authors, without undue reservation. The underlying code for this study is available via Zenodo (https://zenodo.org/doi/10.5281/zenodo.19907832).

## References

[ref1] Abowd J. M. Vilhuber L. (2008). “How protective are synthetic data?” in Privacy in Statistical Databases. Lecture Notes in Computer Science, eds. Domingo-Ferrer J. Saygın Y. (Berlin, Heidelberg: Springer Berlin Heidelberg), 239–246.

[ref2] Bolón-Canedo V. Sánchez-Maroño N. Alonso-Betanzos A. (2013). A review of feature selection methods on synthetic data. Knowl. Inf. Syst. 34, 483–519. doi: 10.1007/s10115-012-0487-8

[ref3] Borrebaeck C. A. K. (2017). Precision diagnostics: moving towards protein biomarker signatures of clinical utility in cancer. Nat. Rev. Cancer 17, 199–204. doi: 10.1038/nrc.2016.153, 28154374

[ref4] Bowles C. Chen L. Guerrero R. Bentley P. Gunn R. Hammers A. . (2018). GAN augmentation: augmenting training data using generative adversarial networks. arXiv. doi: 10.48550/ARXIV.1810.10863

[ref5] Căleanu C. D. Sîrbu C. L. Simion G. (2021). Deep neural architectures for contrast enhanced ultrasound (CEUS) focal liver lesions automated diagnosis. Sensors 21:4126. doi: 10.3390/s21124126, 34208548 PMC8235629

[ref6] Campello C. A. Castanha E. B. Vilardo M. Staziaki P. V. Francisco M. Z. Mohajer B. . (2023). Machine learning for malignant versus benign focal liver lesions on US and CEUS: a meta-analysis. Abdom Radiol 48, 3114–3126. doi: 10.1007/s00261-023-03984-0, 37365266

[ref7] Chauhan V. P. Martin J. D. Liu H. Lacorre D. A. Jain S. R. Kozin S. V. . (2013). Angiotensin inhibition enhances drug delivery and potentiates chemotherapy by decompressing tumour blood vessels. Nat. Commun. 4:2516. doi: 10.1038/ncomms3516, 24084631 PMC3806395

[ref8] Chen R. J. Lu M. Y. Chen T. Y. Williamson D. F. K. Mahmood F. (2021). Synthetic data in machine learning for medicine and healthcare. Nat Biomed Eng 5, 493–497. doi: 10.1038/s41551-021-00751-8, 34131324 PMC9353344

[ref9] Chen Y. Ying C. Binkley M. M. Juttukonda M. R. Flores S. Laforest R. . (2021). Deep learning-based T1-enhanced selection of linear attenuation coefficients (DL-TESLA) for PET/MR attenuation correction in dementia neuroimaging. Magn. Reson. Med. 86, 499–513. doi: 10.1002/mrm.28689, 33559218 PMC8091494

[ref10] Dahmen J. Cook D. (2019). SynSys: a synthetic data generation system for healthcare applications. Sensors 19:1181. doi: 10.3390/s19051181, 30857130 PMC6427177

[ref11] Dewi C. Chen R.-C. Liu Y.-T. Tai S.-K. (2022). Synthetic data generation using DCGAN for improved traffic sign recognition. Neural Comput. & Applic. 34, 21465–21480. doi: 10.1007/s00521-021-05982-z

[ref13] Eisenhauer E. A. Therasse P. Bogaerts J. Schwartz L. H. Sargent D. Ford R. . (2009). New response evaluation criteria in solid tumours: revised RECIST guideline (version 1.1). Eur. J. Cancer 45, 228–247. doi: 10.1016/j.ejca.2008.10.026, 19097774

[ref14] Frid-Adar M. Diamant I. Klang E. Amitai M. Goldberger J. Greenspan H. (2018). GAN-based synthetic medical image augmentation for increased CNN performance in liver lesion classification. Neurocomputing 321, 321–331. doi: 10.1016/j.neucom.2018.09.013

[ref15] Ghosh K. Bellinger C. Corizzo R. Branco P. Krawczyk B. Japkowicz N. (2024). The class imbalance problem in deep learning. Mach. Learn. 113, 4845–4901. doi: 10.1007/s10994-022-06268-8

[ref16] Greenspan H. Van Ginneken B. Summers R. M. (2016). Guest editorial deep learning in medical imaging: overview and future promise of an exciting new technique. IEEE Trans. Med. Imaging 35, 1153–1159. doi: 10.1109/TMI.2016.2553401

[ref17] He K. Zhang X. Ren S. Sun J. (2015). Deep residual learning for image recognition. arXiv. doi: 10.48550/ARXIV.1512.03385

[ref18] Ioffe S. Szegedy C. (2015). Batch normalization: accelerating deep network training by reducing internal covariate shift. arXiv. doi: 10.48550/ARXIV.1502.03167

[ref19] Jain R. K. (2014). Antiangiogenesis strategies revisited: from starving tumors to alleviating hypoxia. Cancer Cell 26, 605–622. doi: 10.1016/j.ccell.2014.10.006, 25517747 PMC4269830

[ref20] Jain R. K. Martin J. D. Stylianopoulos T. (2014). The role of mechanical forces in tumor growth and therapy. Annu. Rev. Biomed. Eng. 16, 321–346. doi: 10.1146/annurev-bioeng-071813-105259, 25014786 PMC4109025

[ref21] Jun S. Shichong Z. Xiao L. Qi Z. Minhua L. Tianfu W. (2016). Stacked deep polynomial network based representation learning for tumor classification with small ultrasound image dataset. Neurocomputing 194, 87–94. doi: 10.1016/j.neucom.2016.01.074, 38826717

[ref22] Kalli M. Mpekris F. Charalambous A. Michael C. Stylianou C. Voutouri C. . (2024). Mechanical forces inducing oxaliplatin resistance in pancreatic cancer can be targeted by autophagy inhibition. Commun Biol 7:1581. doi: 10.1038/s42003-024-07268-1, 39604540 PMC11603328

[ref23] Kalli M. Poskus M. D. Stylianopoulos T. Zervantonakis I. K. (2023). Beyond matrix stiffness: targeting force-induced cancer drug resistance. Trends Cancer 9, 937–954. doi: 10.1016/j.trecan.2023.07.006, 37558577 PMC10592424

[ref24] Kalli M. Stylianopoulos T. (2024). Toward innovative approaches for exploring the mechanically regulated tumor-immune microenvironment. APL Bioeng 8:011501. doi: 10.1063/5.0183302, 38390314 PMC10883717

[ref25] Kondo S. Satoh M. Nishida M. Sakano R. Takagi K. (2023). Ceusia-breast: computer-aided diagnosis with contrast enhanced ultrasound image analysis for breast lesions. BMC Med. Imaging 23:114. doi: 10.1186/s12880-023-01072-9, 37644398 PMC10466705

[ref26] Lango M. Stefanowski J. (2022). What makes multi-class imbalanced problems difficult? An experimental study. Expert Syst. Appl. 199:116962. doi: 10.1016/j.eswa.2022.116962

[ref27] Litjens G. Kooi T. Bejnordi B. E. Setio A. A. A. Ciompi F. Ghafoorian M. . (2017). A survey on deep learning in medical image analysis. Med. Image Anal. 42, 60–88. doi: 10.1016/j.media.2017.07.005, 28778026

[ref28] Liu X. Jiang D. Wang M. Song Z. (2019). Image synthesis-based multi-modal image registration framework by using deep fully convolutional networks. Med. Biol. Eng. Comput. 57, 1037–1048. doi: 10.1007/s11517-018-1924-y, 30523534

[ref29] Liu D. Liu F. Xie X. Su L. Liu M. Xie X. . (2020). Accurate prediction of responses to transarterial chemoembolization for patients with hepatocellular carcinoma by using artificial intelligence in contrast-enhanced ultrasound. Eur. Radiol. 30, 2365–2376. doi: 10.1007/s00330-019-06553-6, 31900703

[ref30] Lu Y. Wu C.-T. Parker S. J. Cheng Z. Saylor G. Van Eyk J. E. . (2022). COT: an efficient and accurate method for detecting marker genes among many subtypes. Bioinform. Adv. 2:vbac037. doi: 10.1093/bioadv/vbac037, 35673616 PMC9163574

[ref31] Lucini F. (2022). The real deal about synthetic data. MIT Sloan Manag. Rev. 63, 11–13.

[ref32] Mahmoud A. Y. Neagu D. Scrimieri D. Abdullatif A. R. A. (2023). Early diagnosis and personalised treatment focusing on synthetic data modelling: novel visual learning approach in healthcare. Comput. Biol. Med. 164:107295. doi: 10.1016/j.compbiomed.2023.107295, 37557053

[ref33] Malone C. D. Fetzer D. T. Monsky W. L. Itani M. Mellnick V. M. Velez P. A. . (2020). Contrast-enhanced US for the interventional radiologist: current and emerging applications. Radiographics 40, 562–588. doi: 10.1148/rg.2020190183, 32125955

[ref34] Mămuleanu M. Urhuț C. Săndulescu L. Kamal C. Pătrașcu A.-M. Ionescu A. . (2023). An automated method for classifying liver lesions in contrast-enhanced ultrasound imaging based on deep learning algorithms. Diagnostics 13:1062. doi: 10.3390/diagnostics13061062, 36980369 PMC10047233

[ref35] Martin J. D. Cabral H. Stylianopoulos T. Jain R. K. (2020). Improving cancer immunotherapy using nanomedicines: progress, opportunities and challenges. Nat. Rev. Clin. Oncol. 17, 251–266. doi: 10.1038/s41571-019-0308-z, 32034288 PMC8272676

[ref36] Mitrea D. Badea R. Mitrea P. Brad S. Nedevschi S. (2021). Hepatocellular carcinoma automatic diagnosis within CEUS and B-mode ultrasound images using advanced machine learning methods. Sensors 21:2202. doi: 10.3390/s21062202, 33801125 PMC8004125

[ref37] Mpekris F. Panagi M. Charalambous A. Voutouri C. Stylianopoulos T. (2024). Modulating cancer mechanopathology to restore vascular function and enhance immunotherapy. Cell Rep Med 5:101626. doi: 10.1016/j.xcrm.2024.101626, 38944037 PMC11293360

[ref38] Mpekris F. Panagi M. Michael C. Voutouri C. Tsuchiya M. Wagatsuma C. . (2023). Translational nanomedicine potentiates immunotherapy in sarcoma by normalizing the microenvironment. J. Control. Release 353, 956–964. doi: 10.1016/j.jconrel.2022.12.016, 36516902

[ref39] Mpekris F. Panagi M. Voutouri C. Martin J. D. Samuel R. Takahashi S. . (2021). Normalizing the microenvironment overcomes vessel compression and resistance to Nano-immunotherapy in breast Cancer lung metastasis. Adv. Sci. 8:2001917. doi: 10.1002/advs.202001917, 33552852 PMC7856901

[ref40] Mpekris F. Voutouri C. Baish J. W. Duda D. G. Munn L. L. Stylianopoulos T. . (2020). Combining microenvironment normalization strategies to improve cancer immunotherapy. Proc. Natl. Acad. Sci. 117, 3728–3737. doi: 10.1073/pnas.1919764117, 32015113 PMC7035612

[ref41] Mpekris F. Voutouri C. Panagi M. Baish J. W. Jain R. K. Stylianopoulos T. (2022). Normalizing tumor microenvironment with nanomedicine and metronomic therapy to improve immunotherapy. J. Control. Release 345, 190–199. doi: 10.1016/j.jconrel.2022.03.008, 35271911 PMC9168447

[ref42] Murphy J. E. Wo J. Y. Ryan D. P. Clark J. W. Jiang W. Yeap B. Y. . (2019). Total neoadjuvant therapy with FOLFIRINOX in combination with losartan followed by Chemoradiotherapy for locally advanced pancreatic Cancer: a phase 2 clinical trial. JAMA Oncol. 5, 1020–1027. doi: 10.1001/jamaoncol.2019.0892, 31145418 PMC6547247

[ref43] Panagi M. Mpekris F. Chen P. Voutouri C. Nakagawa Y. Martin J. D. . (2022). Polymeric micelles effectively reprogram the tumor microenvironment to potentiate nano-immunotherapy in mouse breast cancer models. Nat. Commun. 13:7165. doi: 10.1038/s41467-022-34744-1, 36418896 PMC9684407

[ref44] Panagi M. Voutouri C. Mpekris F. Papageorgis P. Martin M. R. Martin J. D. . (2020). TGF-β inhibition combined with cytotoxic nanomedicine normalizes triple negative breast cancer microenvironment towards anti-tumor immunity. Theranostics 10, 1910–1922. doi: 10.7150/thno.36936, 32042344 PMC6993226

[ref45] Papageorgis P. Polydorou C. Mpekris F. Voutouri C. Agathokleous E. Kapnissi-Christodoulou C. P. . (2017). Tranilast-induced stress alleviation in solid tumors improves the efficacy of chemo- and nanotherapeutics in a size-independent manner. Sci. Rep. 7:46140. doi: 10.1038/srep46140, 28393881 PMC5385877

[ref46] Pezoulas V. C. Zaridis D. I. Mylona E. Androutsos C. Apostolidis K. Tachos N. S. . (2024). Synthetic data generation methods in healthcare: a review on open-source tools and methods. Comput. Struct. Biotechnol. J. 23, 2892–2910. doi: 10.1016/j.csbj.2024.07.005, 39108677 PMC11301073

[ref47] Podell D. English Z. Lacey K. Blattmann A. Dockhorn T. Müller J. . (2023). SDXL: improving latent diffusion models for high-resolution image synthesis. arXiv. doi: 10.48550/ARXIV.2307.01952

[ref48] Polydorou C. Mpekris F. Papageorgis P. Voutouri C. Stylianopoulos T. (2017). Pirfenidone normalizes the tumor microenvironment to improve chemotherapy. Oncotarget 8, 24506–24517. doi: 10.18632/oncotarget.15534, 28445938 PMC5421866

[ref49] Rai H. M. Dashkevych S. Yoo J. (2024). Next-generation diagnostics: the impact of synthetic data generation on the detection of breast cancer from ultrasound imaging. Mathematics 12:2808. doi: 10.3390/math12182808

[ref50] Roth H. R. Lu L. Liu J. Yao J. Seff A. Cherry K. . (2016). Improving computer-aided detection using <?Pub _newline?> convolutional neural networks and random view aggregation. IEEE Trans. Med. Imaging 35, 1170–1181. doi: 10.1109/TMI.2015.2482920, 26441412 PMC7340334

[ref51] Ruiz N. Li Y. Jampani V. Pritch Y. Rubinstein M. Aberman K. (2022). DreamBooth: fine tuning text-to-image diffusion models for subject-driven generation. arXiv. doi: 10.48550/ARXIV.2208.12242

[ref52] Sheridan C. (2019). Pancreatic cancer provides testbed for first mechanotherapeutics. Nat. Biotechnol. 37, 829–831. doi: 10.1038/d41587-019-00019-2, 31375797

[ref53] Shorten C. Khoshgoftaar T. M. (2019). A survey on image data augmentation for deep learning. J. Big Data 6:60. doi: 10.1186/s40537-019-0197-0 PMC8287113 34306963

[ref54] Srivastava N. Hinton G. Krizhevsky A. Sutskever I. Salakhutdinov R. (2014). Dropout: a simple way to prevent neural networks from overfitting. J. Machine Learn. Res. 15, 1929–1958.

[ref55] Stylianopoulos T. Martin J. D. Chauhan V. P. Jain S. R. Diop-Frimpong B. Bardeesy N. . (2012). Causes, consequences, and remedies for growth-induced solid stress in murine and human tumors. Proc. Natl. Acad. Sci. 109, 15101–15108. doi: 10.1073/pnas.1213353109, 22932871 PMC3458380

[ref56] Stylianopoulos T. Munn L. L. Jain R. K. (2018). Reengineering the physical microenvironment of tumors to improve drug delivery and efficacy: from mathematical modeling to bench to bedside. Trends Cancer 4, 292–319. doi: 10.1016/j.trecan.2018.02.005, 29606314 PMC5930008

[ref57] Suganyadevi S. Seethalakshmi V. Balasamy K. (2022). A review on deep learning in medical image analysis. Int. J. Multimed. Inf. Retr. 11, 19–38. doi: 10.1007/s13735-021-00218-1, 34513553 PMC8417661

[ref58] Tajbakhsh N. Shin J. Y. Gurudu S. R. Hurst R. T. Kendall C. B. Gotway M. B. . (2016). Convolutional neural networks for medical image analysis: full training or fine tuning? IEEE Trans. Med. Imaging 35, 1299–1312. doi: 10.1109/TMI.2016.2535302, 26978662

[ref59] Turco S. Tiyarattanachai T. Ebrahimkheil K. Eisenbrey J. Kamaya A. Mischi M. . (2022). Interpretable machine learning for characterization of focal liver lesions by contrast-enhanced ultrasound. IEEE Trans. Ultrason. Ferroelectr. Freq. Control 69, 1670–1681. doi: 10.1109/TUFFC.2022.3161719, 35320099 PMC9188683

[ref60] Varghese B. A. Lee S. Cen S. Talebi A. Mohd P. Stahl D. . (2022). Characterizing breast masses using an integrative framework of machine learning and CEUS-based radiomics. J. Ultrasound 25, 699–708. doi: 10.1007/s40477-021-00651-2, 35040103 PMC9402818

[ref61] Veturi Y. A. Woof W. Lazebnik T. Moghul I. Woodward-Court P. Wagner S. K. . (2023). SynthEye: investigating the impact of synthetic data on artificial intelligence-assisted gene diagnosis of inherited retinal disease. Ophthalmol. Sci. 3:100258. doi: 10.1016/j.xops.2022.100258, 36685715 PMC9852957

[ref62] Voutouri C. Mpekris F. Panagi M. Krolak C. Michael C. Martin J. D. . (2023). Ultrasound stiffness and perfusion markers correlate with tumor volume responses to immunotherapy. Acta Biomater. 167, 121–134. doi: 10.1016/j.actbio.2023.06.007, 37321529

[ref63] Voutouri C. Panagi M. Mpekris F. Stylianou A. Michael C. Averkiou M. A. . (2021). Endothelin inhibition potentiates Cancer immunotherapy revealing mechanical biomarkers predictive of response. Adv. Ther. 4:2000289. doi: 10.1002/adtp.202000289, 41531421

[ref64] Voutouri C. Stylianopoulos T. (2014). Evolution of osmotic pressure in solid tumors. J. Biomech. 47, 3441–3447. doi: 10.1016/j.jbiomech.2014.09.019, 25287111 PMC4256429

[ref65] Voutouri C. Stylianopoulos T. (2018). Accumulation of mechanical forces in tumors is related to hyaluronan content and tissue stiffness. PLoS One 13:e0193801. doi: 10.1371/journal.pone.0193801, 29561855 PMC5862434

[ref66] Wang Q. Junyu G. Wei L. Yuan Y. (2019). Learning from synthetic data for crowd counting in the wild. Proc IEEECVF Conf Comput Vis Pattern Recognit, 8198–8207.

[ref67] Wilson S. R. Greenbaum L. D. Goldberg B. B. (2009). Contrast-enhanced ultrasound: what is the evidence and what are the obstacles? Am. J. Roentgenol. 193, 55–60. doi: 10.2214/AJR.09.2553, 19542395

