# Supplementary material for: Deep learning prediction of chemo-immunotherapy response using tumor perfusion ultrasound images
Source: Front Artif Intell. 2026 Jun 12;9:1805416. doi: 10.3389/frai.2026.1805416 (PMC13303846; doi:10.3389/frai.2026.1805416)
Supplement: Supplementary file 1 [file Data_Sheet_1.PDF]

Supplementary Text

To provide additional context, the proposed CEUS-CNN model was evaluated against established baseline approaches, including transfer learning with ResNet50 and EfficientNetB0 pretrained on the ImageNet dataset.

For fine-tuning, we followed a standard two-stage training strategy. First, we added a custom classification head on top of the pretrained convolutional base and trained only this newly added component while keeping the convolutional base frozen. The rationale for this step is that if the classifier is not already trained, then the error signal propagating through the network during training will be too large and the representations previously learned by the convolutional layers being fine-tuned will be destroyed. Once the classifier was adequately trained, we selectively unfroze the deeper layers of the convolutional base and jointly trained both these layers and the classifier. This approach was adopted, since earlier layers encode more generic features, while deeper layers capture more task-specific representations. Importantly, a very low learning rate was utilized to limit the magnitude of the modifications applied to the pretrained representations.

When these pretrained fine-tuned models were evaluated on the test set, they achieved an accuracy of 0.877, which is lower than the accuracy of 0.930 achieved by our proposed CEUS-CNN model. This comparison demonstrates that our custom architecture achieves improved predictive performance relative to the fine-tuned ResNet50 and EfficientNetB0 models evaluated in this study. The corresponding implementation details and results are available in the provided code.
